# The NPs Role of Assessing and Intervening with Older Adult Drivers

**DOI:** 10.1155/2016/3254857

**Published:** 2016-10-23

**Authors:** Tamatha Arms

**Affiliations:** School of Nursing, University of North Carolina Wilmington, 601 S. College Rd., Office 2034A, Wilmington, NC 28403, USA

## Abstract

As the silver tsunami continues, assessing and intervening with older adult drivers are becoming an essential aspect of the comprehensive geriatric exam. The current lack of time efficient clinical guidelines is a concern and barrier for NPs. The purpose of this study was to identify strategies currently used by NPs. The critical incident technique was used to obtain data from a convenience sample of NPs. A total of 89 incidents were collected. The perspective of the NP can provide important information for developing clinical guidelines to promote individual and community safety.

## 1. Introduction

As the silver tsunami continues through the US and globally, the number of drivers over the age of 75 will increase significantly. As of 2012, there were 36 million older Americans licensed to drive, which was a 34% increase from 1999 [1]. Accidents or unintentional injuries are the 7th leading cause of death in persons 65 and older and are often the result of impairment [2]. In the 64 to 75 age group, injuries from motor vehicle accidents (MVAs) are the leading cause of injury related death and the second leading cause in the 75 to 84 age group [3]. Drivers who are 80 years old and older have 9 times greater fatality rate than drivers aged 25 to 69 [3]. Assessment of driving capability in the older adult is becoming an essential aspect of the comprehensive geriatric exam, even though many primary care providers (PCPs) do not view themselves as experts in this area and may defer driving assessment and capability to the local Department of Motor Vehicles (DMV) office [4]. However, a survey conducted by the American Association of Retired Persons (AARP) showed that 40% of people surveyed aged 50 and older believed that their PCP “could best determine their ability to drive safely” [5, p. 92].

Earlier assessment and intervention lead to prolonged health maintenance and independence, which is an essential aspect that the PCP must consider for the older adult driver. According to the American Association of Nurse Practitioners (AANP) (2012), 80% of nurse practitioners (NPs) practice in a primary care setting. Currently, there are three NP graduates to every one primary care physician graduate in the United States (US), and NPs are improving access to care, especially in rural areas where alternative transportation methods may not be available [6]. Driver safety is considered a public and personal safety issue and the NP has a duty to routinely assess and intervene when appropriate.

Variations across state laws and regulations regarding drivers make it challenging to have a nationally accepted clinical guideline or standard of care specific to assessment of safe driving. Driving regulations, laws, and reporting policies vary according to state and many providers are not fully aware of these laws and policies within the state where they practice. In one state, a healthcare provider can be sued for alerting the DMV of a person's medical diagnosis, whereas, in another state, the healthcare provider is negligible if they do not report it [7]. These extremes in variability make it confusing and difficult for NPs to know what to do and what to follow. Novak et al. [4] report a lack of specific guidelines for primary care providers to use when caring for an older adult driver in the office setting. NPs caring for older adults do not have an efficient evidenced-based screening instrument for use during a routine 15-minute office visit. The current guidelines published by the American Medical Association [8] in collaboration with the National Highway Traffic Safety Administration (NHTSA) are considered “opinion-based” according to one author ([5, 9]). Another source describes the guidelines as comprehensive but lengthy and cumbersome to use during a routine office visit and they are not supported by evidenced-based research [10].

The purpose of this critical incident study was to identify strategies utilized by NPs in the assessment and intervention of older adult drivers in Southeastern North Carolina. The aims of this study were to (1) determine current practice of NP's assessment of and intervention strategies with older adult drivers in Southeastern North Carolina; (2) assess the NPs knowledge deficits in assessing safe driving capability of the older adults; and (3) assess NPs current knowledge of resources available for older adult drivers and their families. Assessment and intervention of safe driving in the older adult will promote health maintenance, independence, and community safety. Healthcare providers have standards of care or published guidelines to assess, treat, and evaluate chronic diseases such as the JNC guidelines for hypertension. A similar guideline for assessment, intervention, and evaluation of driving safety in the older adult would prove beneficial.

## 2. Literature Review

The aging baby boomer generation started turning 65 in 2011 and, by 2030, 1 in every 5 persons will be 65 years old or older in the United States [3]. Persons 85 years old and older are growing at four times the rate of the US population [2]. American's average life expectancy has increased at the same time death rates for the 65 to 84 age group have decreased [11].

As our population ages, there is an increased need for specialized geriatric care that includes a comprehensive, holistic approach. Personal safety behaviors, including safe driving habits, are part of the Shuler Nurse Practitioner Model [12]. This theoretical framework guides the NP to focus on prevention of disease and disability through maintenance of maximal function, improvement in healthcare status, rapidly responding to declines, and periodic routine assessments. The Schuler Model assesses the physiological and the psychological needs and status of the older adult [12]. Through this holistic approach, the NP assesses various areas of functional capability that affect the older adult's ability to drive safely.

Research on NP's driving safety assessment of older adults is scant. A literature review was performed using these databases: EBSCO host, PubMed, Nursing @ ovid, ProAQuest Nursing and Allied Health, Science Direct, and Web of Science. Keywords used were as follows: Older adult, elderly, geriatric, driving, safety, assessment, and Nurse Practitioner. Johnson [13] interviewed 25 NPs on their perceptions of their role in assessing driving ability in the older adult driver who lives in an urban region. Johnson found NPs to be comfortable performing the physical assessment as it relates to driving ability such as vision, hearing, and cognitive status, but they reported being uncomfortable approaching or discussing the topic of driving safety and/or driving termination. Johnson also concluded that early assessment, discussions, and intervention of safe driving were preferred by the older adult, rather than a discussion of driving termination. Even though this study focused on NPs, many other healthcare disciplines such as physicians, social workers, and occupational therapists have a vested interest in driving safety of the older adult. Disciplines outside of healthcare such as law enforcement are equally invested as well.

## 3. Methods

A qualitative research method developed by Flanagan [14], the critical incident technique, was selected as the design for this study. The critical incident technique is a tool used to create a functional description of behavioral activity. According to Flanagan [14], obtaining a description of the activity can be achieved by asking the persons who actually perform the work a series of brief, precise questions aimed at identifying behaviors. Flanagan stressed that, in a critical incident study, the sample size is not determined by the number of participants but rather by the number of critical incidents observed or reported and whether the incidents represent adequate coverage of the activity being studied [15]. The primary objective of the critical incident technique is the development of a behavioral classification system or taxonomy to find solutions to practical problems or to determine the prevalence and distribution of critical behaviors [16]. Critical incident studies have been widely used by health service researchers to identify and categorize behavioral responses of healthcare providers in significant and decisive situations [17, 18].

Critical incident interviews aimed at identifying specific behaviors and strategies used by NPs when assessing and intervening with older drivers were conducted with a sample of 21 NPs recruited through the North Carolina Nurses Association (NCNA) Council of Nurse Practitioners-Coastal Region. The interviews followed the format specified by Flanagan [14]. The open-ended self-administered critical incident survey consisted of the following questions: (1) Think of the last time you cared for an older adult driver in your practice. What assessment strategies or parameters did you use to determine if the driver was safe or not? (2) What strategies did you use to approach that driver about the issue of driving safety? (3) How did you make the determination to intervene if you felt that the older adult was not safe to drive? (4) What barriers did you encounter? Older adult was defined as anyone 65 years old and older for this study.

Permission was obtained through the institutional review board (IRB) at an appropriate institution prior to initiating the study. E-mails were sent out advertising the study and inviting participation.

Data analysis was performed using Statistical Package for the Social Sciences (SPSS, version 20.0) to assess the demographic characteristics of respondents. The narrative data obtained through the critical incident surveys was analyzed through an inductive classification process developed by Flanagan [19]. The critical incidents were initially reviewed by an advanced practice nurse with a specialty in gerontology and an expert in using critical incident methodology to determine which incidents should be included in the analysis. Only incidents that met Flanagan's criteria were included. Incidents that were vague or lacking in detail were discarded. Incidents that were judged to be nearly identical or very similar were grouped together to form subcategories of behaviors. Subcategories were sorted and grouped together to define more inclusive major categories. The incidents were resorted and discussed by the research team to refine and determine the final set of categories. The reliability of the categories was determined through a final sort. Percentages of agreement between the researchers were calculated for each critical incident analysis. As an estimate of the importance of each category, the number of incidents sorted by the researchers was counted and placed into a hierarchical structure or taxonomy that identified categories and their related frequencies.

## 4. Results

### 4.1. Demographic Characteristics

A total of 21 nurse practitioners in the southeast region of the United States (US) participated in this study. The predominantly female sample (*n* = 20, 95%) had a mean age of 48.3 years (SD 11.3 years; range 29–68 years). The average length of employment as a nurse practitioner was 9.1 years (SD 8.4 years). The majority of the participants earned Master's degree (*n* = 18, 85.7%). One held Bachelor's degree with a family nurse practitioner certificate, one had a doctorate of nursing practice (DNP), and one received Ph.D. Approximately one-half of the participants (47.6%) achieved specialty certifications reflecting widely varied areas of advanced nursing practice including: Adult, Geriatrics, Psychiatric Mental Health, Diabetes, Oncology, Hospice, and Wound, Ostomy, and Continence Nursing. None of the study participants had reported attendance at a continuing education program on dealing with older drivers.

Ninety-five percent (*n* = 20) reported that they see older adults (>65 years) who are still driving at least once a week or more often in clinical practice settings. When asked what age they usually start screening for driving ability, 76% (*n* = 16) indicated that there was no specific age level. Two participants (10%) reported initiating driver's safety screening between the ages of 50 and 65 years, while three (*n* = 14%) began screening at the age of 66 years and above. Ninety percent (*n* = 19) involved family members in discussions about driving safety. Six categories of driver behavior prompted NPs to consider performing an evaluation for driving safety. Examples of the circumstances that trigger an assessment of driving capabilities are presented in Table 1.

The participants' self-rating of competence in assessing older driver's safety reflected a moderate level of success at 6.6 (SD 1.3, 1–10 scale with 1* Very Poor* and 10* Excellent*). Self-ratings of comfort in approaching older adults about their driving safety were higher at 8.0 (SD 2.3, 1–10 scale).

## 5. Critical Incident Analysis

### 5.1. Strategies for Assessing Older Driver's Safety

A total of 89 incidents described strategies that nurse practitioners used in assessing an older driver's safety. When the incidents were analyzed, 11 major categories emerged with an interrater agreement of 95%. A listing of assessment categories and circumstances that trigger an assessment of driving capabilities is presented in Table 1.

The largest category,* assessing diminished short-term memory, forgetfulness, or dementia*, accounted for 30 incidents (34%). The use of the mental status exam or the Montreal Cognitive Assessment (MoCA) was mentioned most frequently. Nurse practitioners shared the following:

“I ask them about how many times they are getting lost [while driving]. I also ask them how many times do you ‘beep' your horn at other people, and how many times do other people beep their horns at you? That's a big red flag to me. If they can't tell me how they drove here from their house, that's another red flag.”

“I had one patient whose daughter mentioned a concern about driving. I asked her [the patient] to draw a clock [I used the clock drawing test to assess cognition] and this was really abnormal even though the geriatrician had just done a mental status exam 8 months prior. There was a significant change.”

“I [conducted a] mental status exam. The trigger for me was having to repeat the same conversation over and over again to a patient who was asking the same questions over and over again, day to day, and week to week.”

The second largest category,* Identifying physical frailty,* included 21 or 55% of the incidents (*n* = 21). Participants reported that physical exam findings help determine a patient's driving safety. Specific elements included “conducting a fall assessment,” “assessing [the patient's] ability to turn from side to side, including limited range of motion in the neck,” “impaired reflex timing,” “how well they can feel their feet,” and “assessing whether or not the patient is able to use the dominate foot to control the pedals of the car.” Participants added the following.

“I assess their physical ability on walking by ‘watching them walk in' [to the office] and also look at their ability to get up on the exam table and also their balance.”

“I [look for] patients who describe a poor balance and I assess vertigo or syncope. I also look for people who are losing visual field related to macular degeneration or other visual changes. Most people with this are already limiting their own driving.”

“I observe my patients getting out of and then back into their car. I observe their gait, walking, balance, flexibility, and overall mobility.”

Behavioral incidents in the third largest category,* assessing changes in sensory impairment*, reflect the importance of age-related sensory capacity. Incidents placed in this category primarily related to assessment of visual acuity and hearing. One participant reported that “I ask every patient when they had a last eye exam and will [evaluate their vision] with the Snellen chart.” Another added “if a person cannot seem to hear me in a normal conversation, I'll check their ears for impaction and the whisper test.”

A fourth category,* obtaining collateral information from family members*, accounted for 11% (*n* = 10) of the total number of incidents. As one participant stated that “a trigger is when an adult child makes a comment such as ‘I won't ride with him' or that they will not allow their grandchildren to ride with the patient.”

A fifth category,* assessing for impulsivity/lack of judgment*, accounted for 6% (*n* = 5) of the total number of incidents, while a sixth category contained incidents that described* evaluating medications for drowsiness side-effects* (*n* = 3, 3%). To a far lesser extent, nurse practitioners described* retrieving a self-report of impaired driving or motor vehicle accident* (*n* = 2, 2%),* evaluating symptoms of new onset neurologic disease* (*n* = 2, 2%),* assessing changes in functional status* (*n* = 2, 2%),* assessing history of drugs and/or alcohol abuse* (*n* = 1, 1%), and* evaluating postmyocardial infarction status* (*n* = 1, 1%).

### 5.2. Strategies for Approaching an Older Adult about Driving Safety

A total of 34 incidents were described by nurse practitioner participants. The two most common strategies were* using therapeutic communication to initiate a nonthreatening conversation about driving safety* and* expressing concern for the older adult's driving safely.* The complete list of strategies used is presented in Table 2.

### 5.3. Most Helpful Interventions for Managing Older Driver's Safety

A total of 66 critical incidents were reported which described strategies that nurse practitioners use in practice to increase safety in older adults. When the incidents were analyzed, nine categories of behaviors were determined with an interrater agreement of 100%. Categories included the following:* involving family members, using therapeutic communication, referring patient to occupational/physical therapy, conducting a vision examination, using a step approach to driving termination, using objective data, referring patient to the Department of Motor Vehicles, referring patient to a medical specialist, and limiting medications on the Beers list*. One study has shown that clinicians and older adult drivers are open to the idea of using a tiered approach to driving assessment in the older adult [20]. The taxonomy of nurse practitioner behaviors is presented in Table 3.

The largest category,* involving family members*, accounted for 24% (*n* = 16) of the incidents. As one nurse practitioner stated the following.

“Bring the family into it. Ultimately, they are the ones to take the keys if the patient is resistant.”

Others added the following.

“Sometimes I will voice safety concerns that have been brought up by the family. If the family is present, I try to talk to them and coach them to do the right thing like take the keys or put the care in another place.”

“I tell families to stop being the caregiver and go back to being a family member and make me the ‘bad guy'. I also tell the family to say that ‘I don't want to ride with you' and that is a pretty strong message.”

“It's important to work with families and ask them, ‘Why are you letting the most unable person in the room drive down the street?' It usually has to do with the way this family has made decisions in the past as well as cultural issues, and family dynamics. When I brought up the situation to the family, they agreed and the patient stopped driving until his medical situation improved. He's doing a lot better now.”

The second largest category involved the use of therapeutic communication strategies (*n* = 14, 21%). One participant stated “ask the older adult about how they feel about their ability to drive. This opens up the conversation.” Others added the following.

“I show empathy toward him and his loss of independence and having to rely on family for transportation,” and “I try to be honest about safety concerns.”

“I used therapeutic communication. I told him the concerns such as him not being able to recall the names of the roads that he just drove on to get here.”

“I talked to her conversationally about how she's been doing and how her family has been doing. I started asking about driving when I asked her about her vision and eye glasses. This made her feel comfortable first so then I asked about this [the driving].”

A third largest category contained incidents that described incidents where the nurse practitioner referred the patient to occupational or physical therapy (*n* = 10, 15%), while 12% (*n* = 8) involved conducting a vision screening examination. Eleven percent of the incidents (*n* = 7) described using a step approach to driving termination. Nurse practitioners provided the following examples:

“I use a step approach. First thing is to eliminate driving at night and then I try to find out what's the most important thing they want to drive to. I have one patient who only wants to drive 2 miles from his home to the gas station where he goes into buy his coffee and newspaper and then he drives home. For the longest time this is the only driving he would do and then came the day when I didn't feel he was safe to do that anymore and we had him stop driving…I don't think you can just take it all away from them unless it's really imminent that they are going to hurt themselves.”

“Work with patient to drive only at certain times of the day and to certain areas that are familiar. I had one patient who had very slow response times so we placed a restriction that he could only drive on certain roads and stay off main roads.”

Additional categories reflected the importance of using objective data when discussing driving safety with older adults (*n* = 5, 7%) and referring the patient to the Department of Motor Vehicles (*n* = 3, 5%). Two incidents (3%) described referring the patient to a medical specialist, while one incident (2%) described the importance of avoiding the use of inappropriate, high-risk medications with older adult drivers.

### 5.4. Perceived Barriers

When participants (nurse practitioners) were asked to list perceived barriers for driving cessation, the most frequently mentioned barriers included resistance from the patient with denial, lack of alternative transportation, spousal denial, and increased isolation. A listing of the most frequent barriers that nurse practitioners face in working with older adult drivers is presented in Table 4.

## 6. Discussion

The participants in this study closely represent the national NP demographics. Compared to the 2012 national AANP sample survey, the demographics of NPs nationally were a mean age of 51 years, white (85.4%), Black/African American (3%), female (9.4%), having a graduate level degree (97.2%), and having practiced as a NP for 11 years [21].

### 6.1. Assessment

This study identified strategies used by NPs in the assessment and intervention of older adults in one geographical region. Obvious limitations of this study are the limitation to one geographical area. To the author's knowledge, this is one of the first studies to examine self-report behaviors and strategies of NPs on this topic. Clinical practice for assessing and intervening with older adult drivers varies significantly from provider to provider. The significant public safety aspect of this topic needs to be highlighted. NPs are educated to practice holistically and place great importance on building rapport and trust with their patients and their families. This trusting relationship will prove beneficial when a difficult conversation such as driving cessation is needed.

### 6.2. Intervention

Impaired cognition was the most common reason a NP determined a need to intervene. Assessing cognitive status, physical frailty, and sensory impairments were the most common physical exam strategies used. Obtaining collateral information from family members ranked just below obtaining physical exam or objective data. Using therapeutic communication techniques and a concern of safety for the older adult were the most common strategies used in approaching the older adult about driving. NPs ranked the most helpful intervention was to involve family members and the least helpful intervention was the lack of family guidance from the provider. Dealing with patient concerns about lack of transportation, especially in rural areas, and resistance to driving termination were the two most common perceived barriers. The wide variation in ways which NPs make the determination to address driving in older adults and the lack of consistent guidelines for dealing with this issue should be noted.

## 7. Clinical Implications

As the supply of primary care physicians is expected to decrease, the number of nurse practitioners (NPs) is expected to increase 94% between 2008 and 2025. NPs are trained to provide a holistic approach to patient care and approximately half of all NPs practice in the primary care setting [22].

Standardized curriculum for nurse practitioner students does not require faculty to address concerns of older adult drivers; therefore, few NPs graduate with this training. This could be due to lack of clinical guidelines, and faculty are unclear as to what should be taught. As this is a public safety concern, it should become part of standardized curriculum for the NP student. Having difficult conversations with older adults such as driving cessation is time consuming and possibly threatens the patient-provider relationship. There is a need for the development of clinical guidelines that NPs can use efficiently during a routine office visit. NPs feel comfortable using evidenced-based clinical guidelines to guide their clinical practice with common chronic disease management. Development of an evidenced-based clinical guideline for assessment and intervention with older adult drivers would be used similarly.

Our social work and occupational therapist colleagues have been working on this issue for quite some time. Occupational therapists have developed guidelines for them to use in assessment of older adult drivers. NPs and NP students should also be aware of community resources available in their areas of clinical practice. Simply making themselves aware of resources available to them and the older adult, such as the online AARP driver safety program that can be found at http://www.aarp.org/home-garden/transportation/driver_safety/ [23] would prove beneficial.

## 8. Conclusion

This study shows the varied and inconsistent clinical practice of NPs assessing and intervening with older adults drivers. Who is to say that one strategy is better than the other? Much further research would need to be conducted to reach this conclusion. It is clear that NPs value the relationship they have with their patients and they value the need to intervene when public safety is at risk. Development of evidenced-based clinical guidelines would prove beneficial for NPs to use in clinical practice, as long as the guidelines were able to be used quickly and efficiently.

## Figures and Tables

**Table 1:** Major categories of nurse practitioner strategies for determining when to intervene with an older driver (*n* = 89 incidents).

Major category	Number of incidents	Percent
*(I) Identifying changes in physical status (38 incidents, 43%)*		
(1) Identifying physical frailty (decreased mobility and strength)	21	55%
(2) Assessing changes in sensory impairment	12	32%
(3) Evaluating symptoms of new onset neurologic disease	2	5%
(4) Assessing changes in patient's functional status	2	5%
(5) Evaluating postmyocardial infarction status	1	2%
*(II) Evaluating older driver's mental status (35 incidents, 39%)*		
(1) Assessing diminished short-term memory, forgetfulness, or diagnosis of dementia	30	86%
(2) Assessing for impulsivity/lack of judgment	5	14%
*(III) Hearing concerns from family members (10 incidents, 12%)*		
(1) Obtaining collateral information from family members	10	100%
*(IV) Evaluating medications (3 incidents, 3%)*		
(1) Evaluating medications for drowsiness side-effects	3	100%
*(V) Retrieving driving information (2 incidents, 2%)*		
(1) Obtaining a self-report of impaired driving	1	50%
(2) Retrieving a report of a motor vehicle accident	1	50%
*(VI) Assessing drug and alcohol abuse (1 incident, 1%)*		
(1) Assessing history of drugs and/or alcohol abuse	1	100%

**Table 2:** What strategies did you use to approach the topic of driving safety with the older adult (*n* = 34)?

Strategies for approaching an older adult about driving safety	*N* (%)
Use therapeutic communications strategies to initiate a nonthreatening conversation about safe driving	6 (18%)
Express a concern for the older adult's driving safety	6 (18%)
Present objective exam data to support the discussion	5 (15%)
Present patient education about safe driving	4 (12%)
Talk to family members first	4 (12%)
Use an honest, empathetic approach	2 (6%)
Be blunt with the patient	2 (6%)
Avoid the topic during a “sick visit”	2 (6%)
Do not bring up the topic on an initial visit but wait until a relationship has been established	1 (3%)
Ask the older adult to describe how he/she drove to the current appointment	1 (3%)
Use motivational interviewing techniques	1 (3%)

**Table 3:** Most helpful interventions for managing older driver safety (*n* = 66).

Most helpful interventions	*N* (%)
Involving family members	16 (24%)
Using therapeutic communication with the patient	14 (21%)
Referring patient to occupational therapy/physical therapy	10 (15%)
Conducting a vision examination	8 (12%)
Using a step approach to driving termination	7 (11%)
Using objective data	5 (7%)
Referring patient to the Department of Motor Vehicles	3 (5%)
Referring patient to a medical specialist (psychiatrist, neurologist)	2 (3%)
Limiting medications on the Beers list	1 (2%)

**Table 4:** Most frequently encountered barriers (*n* = 46).

Barriers	*N* (%)
Dealing with patient concerns about lack of transportation	23 (50%)
Encountering patient resistance to give up a driver's license	16 (35%)
Identifying cognitive impairments that limit an older driver's ability to understand the need to stop driving	4 (9%)
Dealing with an older driver's fear of increasing isolation which may result in depression, anxiety, and increased substance abuse	2 (4%)
Dealing with a spouse who allows an older driver with dementia to continue driving	1 (2%)
